# Whole genome sequencing and metabolomics analyses reveal the biosynthesis of nerol in a multi-stress-tolerant *Meyerozyma guilliermondii* GXDK6

**DOI:** 10.1186/s12934-020-01490-2

**Published:** 2021-01-03

**Authors:** Xueyan Mo, Xinghua Cai, Qinyan Hui, Huijie Sun, Ran Yu, Ru Bu, Bing Yan, Qian Ou, Quanwen Li, Sheng He, Chengjian Jiang

**Affiliations:** 1 State Key Laboratory for Conservation and Utilization of Subtropical Agro-Bioresources, Guangxi Research Center for Microbial and Enzyme Engineering Technology, College of Life Science and Technology, Guangxi University, Nanning, 530004 China; 2 Guangxi Key Lab of Mangrove Conservation and Utilization, Guangxi Mangrove Research Center, Guangxi Academy of Sciences, Beihai, 536000 China; 3 Guangxi Birth Defects Prevention and Control Institute, Maternal and Child Health Hospital of Guangxi Zhuang Autonomous Region, Nanning, 530033 China

**Keywords:** *Meyerozyma guilliermondii*, Whole genome sequencing, Metabolomics technology, Aroma-producing mechanism, Biosynthesis

## Abstract

**Background:**

Nerol (C_10_H_18_O), an acyclic monoterpene, naturally presents in plant essential oils, and is used widely in food, cosmetics and pharmaceuticals as the valuable fragrance. Meanwhile, chemical synthesis is the only strategy for large-scale production of nerol, and the disadvantages of chemical synthesis greatly limit the production and its application. These defects drive the interests of researchers shift to the production of nerol by eco-friendly methods known as biosynthesis methods. However, the main technical bottleneck restricting the biosynthesis of nerol is the lacking of corresponding natural aroma-producing microorganisms.

**Results:**

In this study, a novel multi-stress-tolerant probiotics *Meyerozyma* *guilliermondii* GXDK6 with aroma-producing properties was identified by whole genome sequencing and metabolomics technology. GXDK6 showed a broad pH tolerance in the range of 2.5–10.0. The species also showed salt tolerance with up to 12% NaCl and up to 18% of KCl or MgCl_2_. GXDK6 exhibited heavy-metal Mn^2+^ tolerance of up to 5494 ppm. GXDK6 could also ferment with a total of 21 kinds of single organic matter as the carbon source, and produce abundant aromatic metabolites. Results from the gas chromatography–mass spectrometry indicated the production of 8–14 types of aromatic metabolites (isopentanol, nerol, geraniol, phenylethanol, isobutanol, etc.) when GXDK6 was fermented up to 72 h with glucose, sucrose, fructose, or xylose as the single carbon source. Among them, nerol was found to be a novel aromatic metabolite from GXDK6 fermentation, and its biosynthesis mechanism had also been further revealed.

**Conclusion:**

A novel aroma-producing *M. guilliermondii* GXDK6 was identified successfully by whole genome sequencing and metabolomics technology. GXDK6 showed high multi-stress-tolerant properties with acid–base, salty, and heavy-metal environments. The aroma-producing mechanism of nerol in GXDK6 had also been revealed. These findings indicated the aroma-producing *M. guilliermondii* GXDK6 with multi-stress-tolerant properties has great potential value in the fermentation industry. 
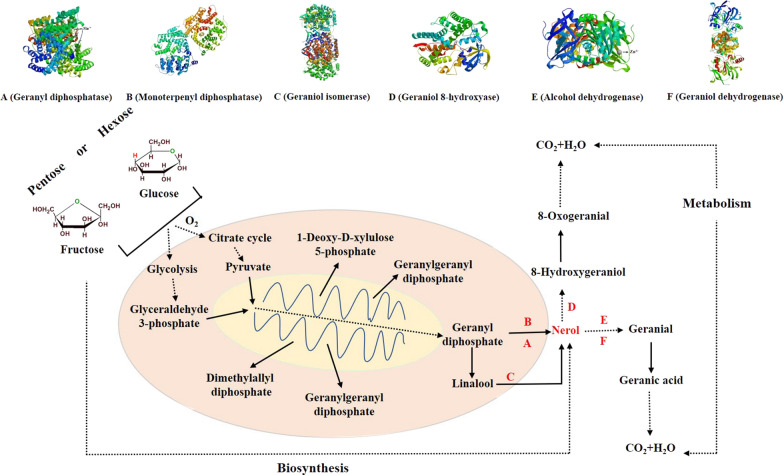

## Background

Nerol (C_10_H_18_O), an acyclic monoterpene, naturally presents in plant essential oils and is used widely in food, cosmetics and pharmaceuticals as the valuable fragrance [1, 2]. Meanwhile, nerol possesses antifungal activity against *Aspergillus niger* [3] and *Aspergillus flavus* [4] and it can be used as a potential antifungal agent for food preservation. The global annual demand for nerol is more than 5,000 tons per year, but the global production capacity of nerol is only about 3,000 tons, which is still in greater demand for improvement. At present, chemical synthesis is the only way for large-scale production of nerol, but the disadvantages of chemical synthesis such as high cost, complicated process, serious pollution, low yield, and more by-products greatly limit the production and its application. These defects drive the interests of researchers shift to the production of nerol by eco-friendly methods, which are often known as biosynthesis methods. However, the main technical bottleneck restricting the biosynthesis of nerol is the lacking of corresponding natural aroma-producing microorganisms, especially the aroma-producing probiotics.

Aroma-producing probiotics has been demonstrated to be greatly beneficial to hosts and ecological balance. In the past few decades, well-known aroma-producing probiotics had been identified with yeasts, *Lactobacillus*, and *Bacillus subtilis* [5]. These species can produce abundant beneficial metabolites through their own metabolic regulations, which showed extremely high practical application. However, most probiotics exist in specific environments, resulting in the interspecies variations among the aroma substances often produced by different probiotics. Most probiotics cannot be fermented for a long time because of the unfavorable conditions, such as the acidification of carbohydrates [6].

To date, known aroma-producing probiotics are generally condition-dependent microorganisms [7]. Few probiotics can ferment different nutrient substrates and generate beneficial aromatic metabolites under special environments (e.g., high salt, extremely strong alkaline, or strong acidic condition), and sustain prolonged aroma production [8, 9]. The screening of aroma-producing probiotics with multistress tolerance has become increasingly urgent [10].

*M. guilliermondii* is known for the production of flavour compounds in fermented food products, has been widely used in food fermentation in recent years [11, 12]. For example, Coda et al. [11] used *M. guilliermondii* to ferment wheat bread, results showed that many active compounds could be synthesized by *M. guilliermondii*, and the shelf life of wheat bread was effectively extended by 14 days. *M. guilliiermondii* had also been demonstrated to belong to a kind of probiotic with great potential, which can produce various health bioactive compound, such as the efficient production of isoflavone aglycone [13, 14], the overproduction of vitamin B2 (riboflavin) [15]. Herein, a novel aroma-producing *M. guilliermondii* GXDK6 with multistress-tolerant properties was isolated and identified from subtropical mangrove sediments mainly on the basis of whole genome sequencing. Metabolomics technology based on gas chromatography–mass spectrometry (GC–MS) was also performed to investigate the production of aroma. Among the aromatic productions, nerol was found as a novel aromatic metabolite from GXDK6 fermentation, and its biosynthesis mechanism had also been further revealed. To the authors’ knowledge, *M. guilliermondii* GXDK6 is the first multi-stress-tolerant probiotics from subtropical marine mangrove sediments. This research shows the novel strategies of natural biosynthesis of nerol and also provides new materials and theoretical references for the industrial production of aroma-producing species.

## Results and discussion

### Physicochemical characterization of GXDK6

**Fig. 1:**
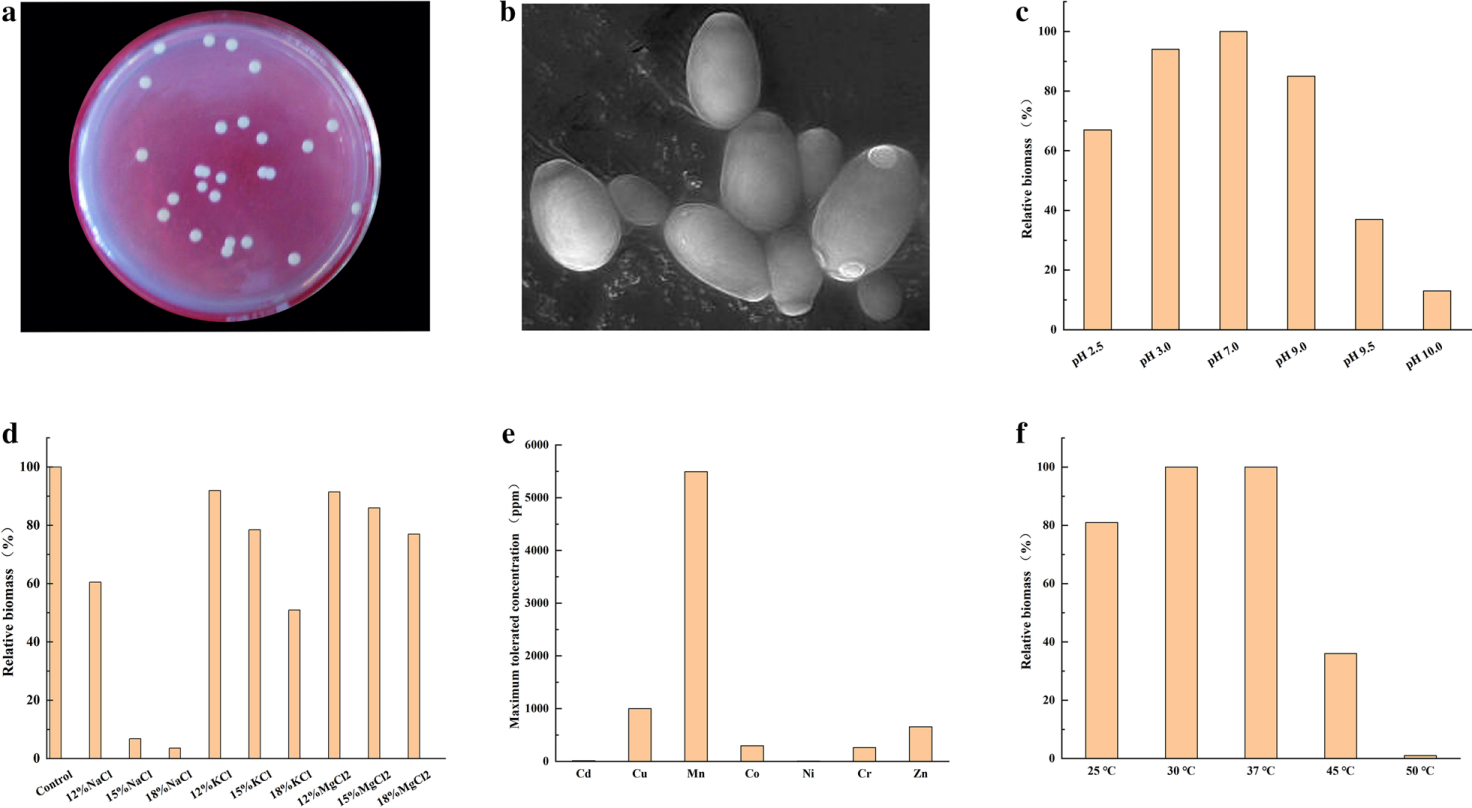
Physicochemical characteristics of *M. guilliermondii* GXDK6.** a** Colony characteristics; **b** Morphology of GXDK6 as observed under a scanning electron microscope; **c** The relative biomass of GXDK6 in the pH range of 2.5–10.0; **d** The relative biomass of GXDK6 under gradually increasing salt concentration; **e** The relative biomass of GXDK6 under different heavy metals; **f** The relative biomass of GXDK6 in the temperature range of 25–50 °C

As shown in Fig. 1a, the colony of GXDK6 was milky white, round, convex, and smooth. The SEM results showed that GXDK6 was similar to the typical yeast species of *Pichia anomala* Y197-13 [16]. In addition, the cell size was between 2–12 μm, and the cell morphology was oval or semi-oval with a smooth surface (Fig. 1b).

### Multi-stress-tolerant properties of GXDK6

As shown in Fig. 1c, GXDK6 could be incubated continuously in the pH range from 2.5 to 10.0. This result indicated that GXDK6 had a strong resistance to acids and alkalis. However, when GXDK6 was incubated in an acidic condition at pH 2.5 or pH 3.0, the relative biomass GXDK6 was higher than that at pH 9.5 or 10.0, respectively. This result suggested that GXDK6 showed better resistance to an acidic environment.

The salt-tolerance results of GXDK6 indicated that the species was a probiotic with tolerance to high-salt concentrations (Fig. 1d). GXDK6 could be incubated continuously up to 12% NaCl, with the relative biomass of approximately 10% with that in 1% NaCl, and up to 18% KCl or MgCl_2_, with the relative biomass of near 90% with that with no salt condition. These findings indicated that the growth of GXDK6 decreased drastically with increasing concentration of NaCl up to 12% but showed no significant impact with increasing concentration of KCl or MgCl_2_ up to 18%. As shown in Fig. 1e, GXDK6 also showed good tolerance to the seven heavy metals (i.e., Cd^2+^, Cu^2+^, Mn^2+^, Co^2+^, Ni^2+^, Cr^2+^, and Zn^2+^). The maximum tolerated concentration for Ni^2+^ was 5.8 ppm, while Mn^2+^ was 5494 ppm. The tolerance for Cu^2+^ was also more than 1000 ppm, but the maximum tolerance for Co^2+^, Cr^2+^, and Zn^2+^ was more than 250 ppm. This characteristic shows the potential of the species for the deodorization and bioremediation in heavy metal environments. Similar results were reported by Fernández et al. [17] and Bhakta et al. [18]. The temperature sensitivity of GXDK6 was also investigated (Fig. 1f). GXDK6 could be incubated at 25–45 ℃, and the optimal incubation temperature was 30–37 ℃. The relative biomass decreased drastically to 25.22% at 45 ℃ compared with that at 30 ℃. In addition, when the incubation temperature increased to 50 ℃, GXDK6 stopped to reproduce. A similar result was reported by Ndubuisi et al. [19], who found that the growth of heat-resistant *Pichia* was significantly inhibited by increasing temperature, and the ethanol production efficiency of *Pichia* was lowered.

### Whole genome sequencing analysis of GXDK6

**Fig. 2:**
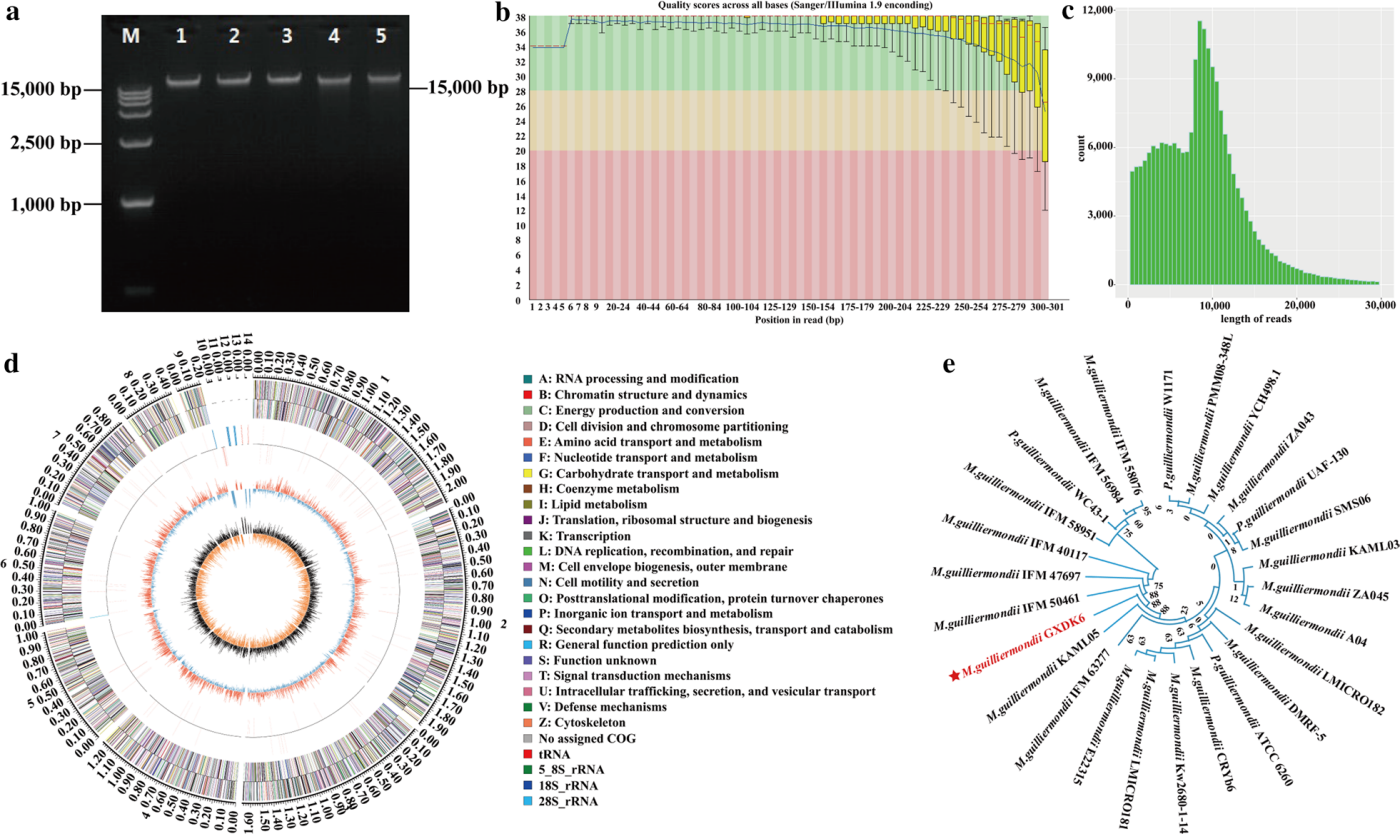
Identification and whole genome sequencing analysis of *M. guilliermondii* GXDK6. **a** The genomic DNA verification map of the *Pichia* strain; **b** Quality scores of the *Pichia* strain across all bases; **c** The sequence length distribution of the third-generation sequencing data; **d** The spliced genome information of the *Pichia* strain; **e** The phylogenetic tree of the *Pichia* strain

The whole genomic sequencing of GXDK6 was performed using the whole genome shotgun method reported by Marcel et al. [20]. The number of reads of GXDK6 was 8, 319, 572 items, the total bases were 2, 477, 073, 673 bps, of which the GC content accounted for 38.88% (Additional file 1). The fuzzy bases were only 0.001%, Q_20_% was 94.60%, and Q_30_% was 86.73%. Genome of GXDK6 was ~ 15,000 bps. The results showed that the extracted DNA was a clear single band, indicating that the extracted DNA was suitable for subsequent analysis (Fig. 2a). Then, a single-base mass distribution map of the sequencing results was constructed (Fig. 2b). The abscissa is the base position of the reads (5ʹ–3ʹ), while the ordinate is the base Q value statistics of all reads. The red and blue lines represent the median and average value of the reads. The yellow line shows that the reads are in the 25% –75% interval, and the tentacles represent that the reads are in the 10–90% interval. The results showed that the bases in the middle had higher base quality. The average quality of the data filtered was also reliable (Fig. 2c).

The sequence length distribution of the third-generation sequencing data is also shown in Fig. 2d, which was mainly used to reflect the average mass distribution of the sequencing data [21]. From Fig. 1e, the whole genome sequence length was good, while the ratio of the uncertain bases to the length of the splicing sequence was 0. This result indicated that the sequence could be used for subsequent gene splicing.

Sequencing and analysis of the ITS domain indicated that the strain with higher consistency basically belong to *Meyerozyma* sp. The evolutionary distance between GXDK6 and other species has been showed in the species evolutionary tree clearly (Fig. 2e). GXDK6 has a high affinity with 88% confidence level with *M. guilliermondii* KAML05, *M. guilliermondii* IFM6377, etc., instead of *P. guilliermondii* ATCC6260. Therefore, GXDK6 was classified as yeast *Meyerozyma guilliermondii*.

A complete sequence comparison of the genome sequences was conducted by using the online software BUSCO (http://busco.ezlab.org, v3.0.2) to obtain the percentage of single-copy genes in the total single-copy genes [22]. As shown in Fig. 2e, the spliced genome data of the species were relatively complete. According to the alignment results, GXDK6 belonged to the *Pichia* genus of the *Saccharomyces* family and showed the closest relation to *M. guilliermondii*. Therefore, the yeast *Pichia* was further confirmed as *M. guilliermondii* GXDK6.

### Ability for single-organic matter fermentation

**Fig. 3:**
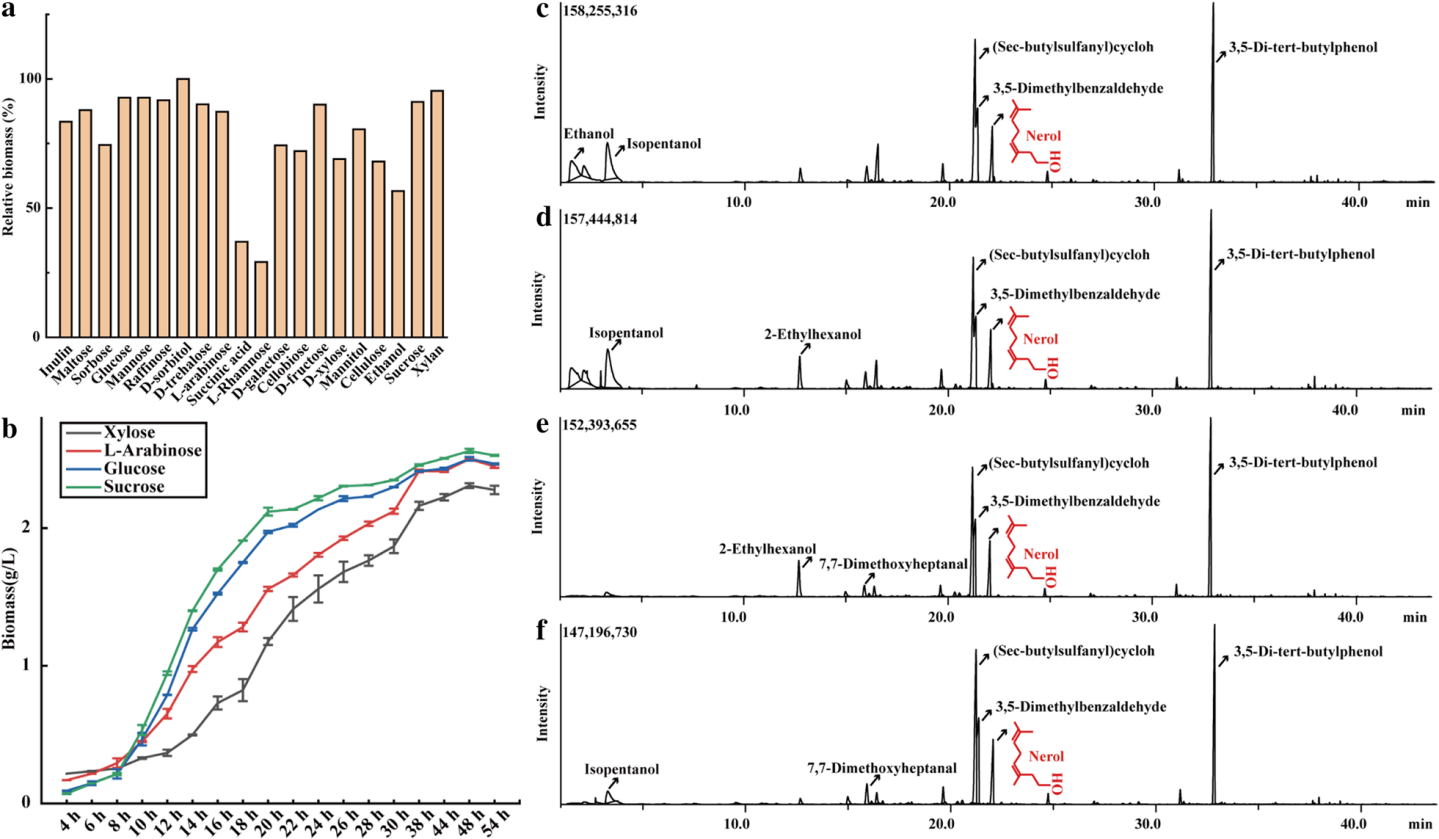
Ability of GXDK6 for organic matter fermentation. **a** Relative biomass of *M. guilliermondii* GXDK6 under the different single-carbon sources; (b) Growth curve of GXDK6 with xylose, L-arabinose, glucose and sucrose as the single-carbon source, respectively; **c** The peak spectrum of GXDK6 fermented with glucose; **d** The peak spectrum of GXDK6 fermented with sucrose; **e** The peak spectrum of GXDK6 fermented with fructose; **f** The peak spectrum of GXDK6 fermented with xylose

As shown in Fig.  3a, 21 organic matters (i.e., glucose, sucrose, fructose, xylose, xylan, sorbitol, raffinose, mannose, trehalose, cellulose, maltose, arabic candy, inulin, mannitol, sorbose, D-galactose, cellobiose, wheat bran, ethanol, succinic, and L-rhamnose) as the sole carbon source could be fermented by GXDK6. This result indicated that GXDK6 showed a strong ability to utilize organic matter, such as pentose and hexose.

Figure 3b showed that the GXDK6 grew best when used with sorbitol as the sole carbon source, with a dry cell weight of ~ 1.499 g/L, but grew slowest with L-rhamnose, with a dry cell weight of ~ 0.437 g/L. Therefore, the growth rate results showed significant differences in the rate by which GXDK6 utilized diverse organic matters. The order of utilization could be summarized as follows: sorbitol > xylan > raffinose > mannose > sucrose > fructose > trehalose > inulin > maltose > arabic candy > mannitol > glucose > sorbose > D-galactose > cellobiose > xylose > cellulose > ethanol > wheat bran > succinic > L-rhamnose.

Many types of aroma-producing yeasts, such as *Hansenula*, *Candida*, *S. cerevisiae*, *Pichia pastoris*, and *Sphaeropsis sphaeroides*, had been reported [23]. The different yeasts also presented diverse aroma-producing characteristics, such as floral fragrance, fruity, delicateness, sweetness, and wine aroma. However, an aroma-producing yeast that can ferment various organic matters, produce abundant aromatic beneficial metabolites, and possess a strong multi-stress tolerance to various environments has not been reported yet. Therefore, *M. guilliermondii* GXDK6 will be a potential probiotic with important application value.

The metabolites produced by GXDK6 with glucose, sucrose, fructose, or xylose as sole carbon sources was detected with GC–MS method, results showed that 14, 20, 16, and 26 peaks were detected in the samples (Fig. 3a–d). Among them, when GXDK6 was fermented with six carbon sugars (glucose, sucrose) as a single carbon source for 72 h, the top five substances with peak areas were 3,5-ditert-butylphenol, sec-butyl cyclohexyl sulfide, isoamyl alcohol, 3,5-dimethylbenzaldehyde, and ethanol, respectively. However, when GXDK6 was fermented for 72 h with pentose (fructose, xylose) as a single carbon source, the top five substances in peak area were 3,5- ditert-butylphenol, sec-butyl cyclohexyl sulfide, 3,5-dimethylbenzaldehyde, nerol, and isoamyl alcohol (or 2-ethyl hexanol), respectively. Thus, GXDK6 could ferment with different kinds of organic matter as the sole carbon source to produce aromatic metabolites. However, the distinct metabolic regulation networks and mechanisms remain unknown [24, 25], which still need further study.

### Metabolomic analysis of GXDK6

**Fig. 4:**
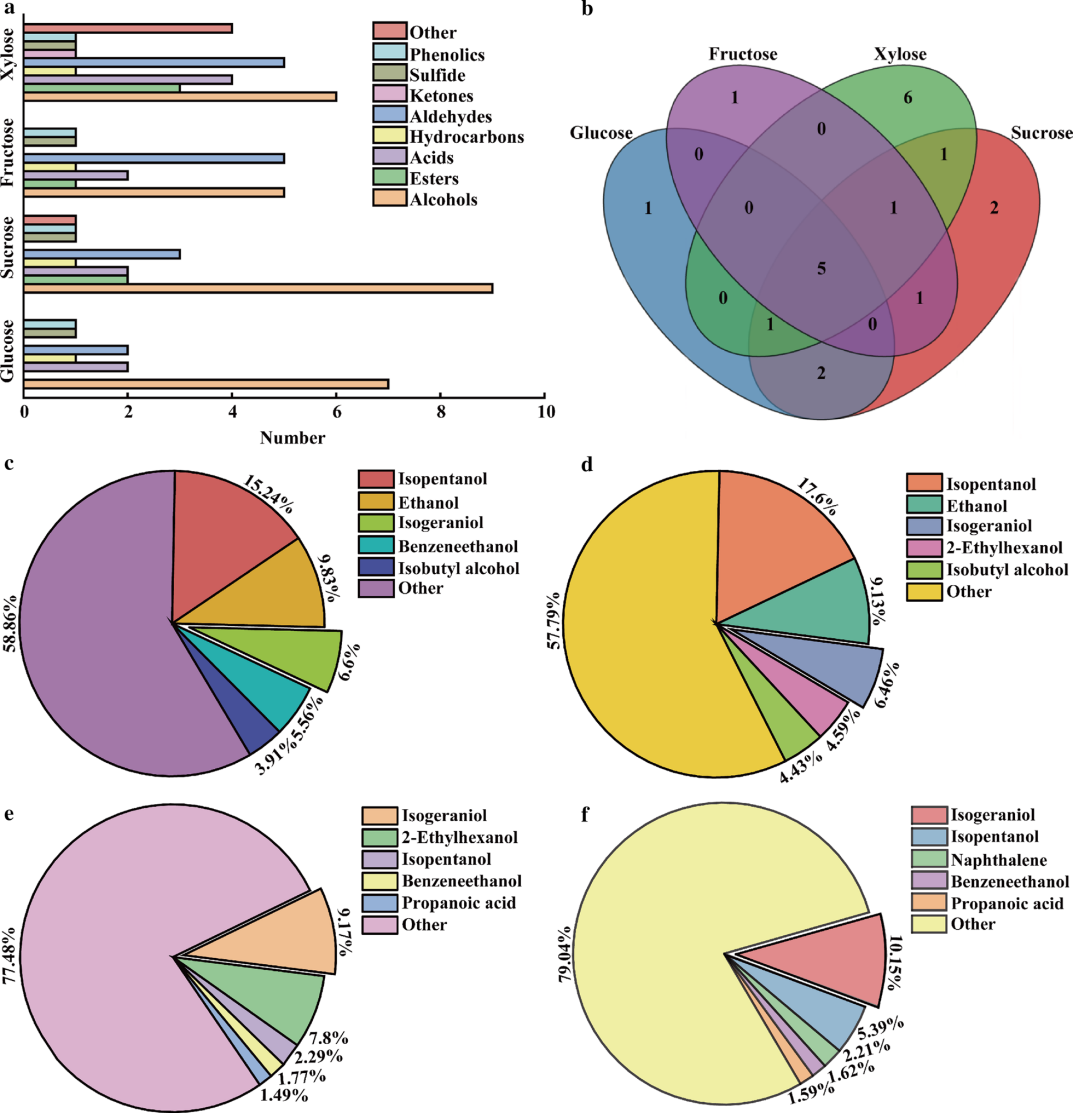
Metabolomic analysis of GXDK6 in fermenting different carbon sources. **a** Classification of the metabolites; **b** Wayne diagram analysis of the aromatic metabolites; **c** Top 5 aromatic metabolites of GXDK6 fermented with glucose; **d** Top5 aromatic metabolites of GXDK6 fermented with sucrose; **e** Top 5 aromatic metabolites of GXDK6 fermented with fructose; **f** Top 5 aromatic metabolites of GXDK6 fermented with xylose

As shown in Fig. 4, the metabolites of GXDK6 produced from the selected carbon sources can be classified as alcohols, esters, acids, hydrocarbons, aldehydes, ketones, sulfide, phenolics, and other organic substance (Fig. 4a). However, alcohols, lipids and organic acids are usually defined as the main aromatic metabolites. Based on this, the main aromatic metabolites produced by GXDK6 fermentation of glucose and sucrose are 9 and 13 types, respectively (Table 1), and the aromatic metabolites produced by fermentation of fructose and xylose are 8 and 14 types, respectively (Table 1).

**Table 1:** Aromatic metabolites of GXDK6 fermented with inconsistent organic matter

Organic matter	Aromatic metabolites	Percentage of total metabolites (%)
Glucose	Isopentanol, Ethanol, Nerol, Phenylethanol, Isobutanol, 2-ethylhexyl alcohol, propionic acid, formic acid, 1-methoxy-2-propanol	45.79%
Sucrose	Isopentanol, Ethanol, Nerol, 2-ethy-hexanol, Isobutanol, Phenylethanol, propionic acid, formic acid, Dibutyl phthalate, α—terpineol, 1-methoxy-2-propanol, Cyclopentyl 4-ethylbenzoate, Farnesol	48.67%
Fructose	Nerol, 2-ethylhexanol, Isopentanol, Phenylethanol, propionic acid, formic acid, α—terpineol, Dibutyl phthalate	24.82%
Xylose	Nerol, Isopentanol, Phenylethanol, propionic acid, formic acid, 2-ethylhexyl alcohol, α—terpineol, Diisobutyl phthalate, acetic acid, Dimethylsiloxanediol, Methyl 4-ethylbenzoate, Farnesol, Butyl isobutyl phthalate, 3-ethoxypropionic acid	23.13%

Among these aromatic metabolites, no matter which carbon source is used for fermentation, there are five aromatic metabolites which are the same (Fig. 4b), namely isoamyl alcohol, nerol, phenethyl alcohol, formic acid, and propionic acid, respectively. These results indicate that GXDK6 has the same metabolic pathway when fermenting five-carbon sugar and six-carbon sugar. In order to further studying the difference of aroma production from inconsistent carbon sources of GXDK6 fermentation, the top five aromatic metabolites were selected according to the percentage of content. Among them, isopentanol (15.24, 17.60%), ethanol (9.83, 9.13%), nerol (6.60, 6.46%), isobutanol (9.83, 9.13%), and 2- ethylhexanol (9.83, 9.13%) are the top five aromatic metabolites of six-carbon sugar (glucose and sucrose) fermented by GXDK6. Among the aromatic metabolites of five-carbon sugars (fructose and xylose) fermented by GXDK6, nerol (9.17, 10.15%), isopentanol (2.29, 5.39%), phenethyl alcohol (1.77, 1.62%), formic acid (1.09%, 1.23%), 2- ethylhexanol (1.09%, Fig. 4e) or propionic acid (1.49%) ranked in the top five (Fig. 4f). These results suggested that GXDK6 showed distinct fermentation abilities with different carbon sources as substrates. However, this species could also produce partial similar beneficial aromatic metabolites, which could be due to its diverse mechanisms in fermenting organic matters [26, 27].

### Metabolic pathways of nerol and its biosynthesis mechanism

**Fig. 5:**
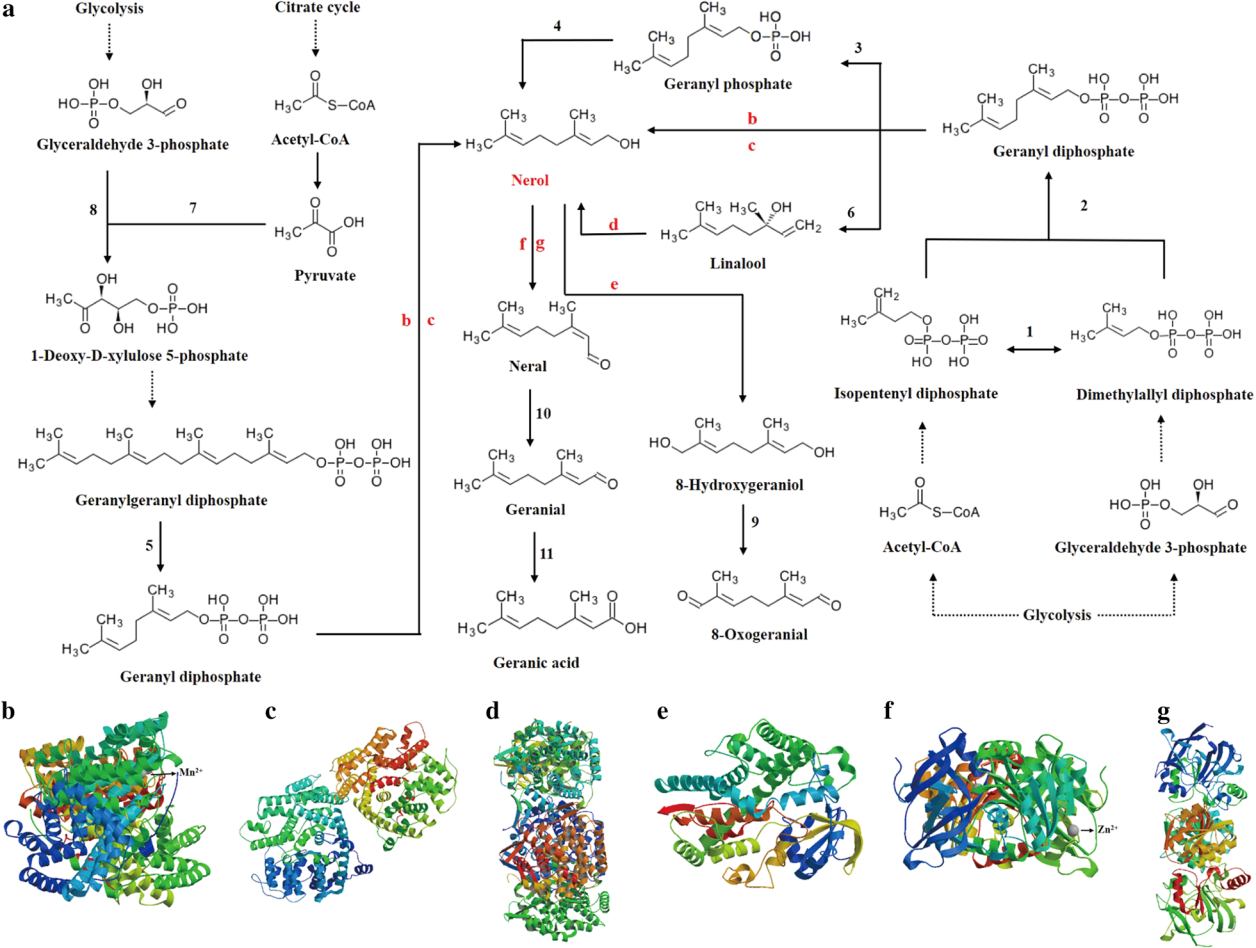
The metabolic pathway of nerol and its relevant regulatory proteins in GXDK6. **a** Metabolic pathway of nerol; **b** Geranyl diphosphatase; **c** Monoterpenyl diphosphatase; **d** Geraniol isomerase; **e** Geraniol 8-hydroxyase; **f** Alcohol dehydrogenase; **g** Geraniol dehydrogenase

In order to further study the molecular mechanism of aroma production by GXDK6 fermentation, nerol (Additional file 2) was taken as a representative novel aromatic metabolite from *M. guilliermondii*, which was further compared and analyzed with the whole genome data of GXDK6 (Additional file 3), and the metabolic pathway was further elucidated. As shown in Fig. 5, the upstream sources of glycolysis or citric acid cycle 1-deoxy-d-xylulose5-phosphate geranyl diphosphate or linalool nerol, was firstly converted into final geranic acid or 8-Oxogeranial. In this process, the proteins involved in nerol synthesis were geranyldi phosphotase, monoterphenyl diphosphatase, and geraniol isomerase. The proteins involved in nerol metabolic transformation were geraniol 8-hydroxyase, alcohol dehydrogenase, and geraniol dehydrogenase. As reported by Zong et al. [10], nerol was biosynthesized in the metabolic engineered *Escherichia coli* from glucose with an accumulation of 0.053 ± 0.015 mg/L, and the biosynthesis mechanism had also been revealed. The truncated neryl diphosphate synthase gene tNDPS1 was expressed that catalyzed isopentenyl diphosphate (IPP) and dimethylallyl diphosphate (DMAPP) form neryl diphosphate (NPP), and then the nerol synthase gene GmNES was co-expressed to synthesize the final product nerol from NPP. However, the accumulation of nerol of GXDK6 was ~ 2.740 mg/L (*p* < 0.05) when fermented with glucose as the substrate, which is higher than it was produced by *E.coli* fermentation [10], and the biosynthesis mechanism of nerol in native *Meyerozyma guilliermondii* has not been reported yet.

The structure and function of the six proteins were further investigated (Fig. 5), results showed that geranyl diphosphatase was a dimer protein with ~ 65 kDa, and its corresponding ligand was found as Mn^2+^ (Fig. 5b), which suggested that geranyl diphosphatase could be bound and interacted with Mn^2+^, and promoted the catalytic reaction to produce more nerol [28]. Monoterpenyl diphosphatase was also a dimer protein with a molecular weight ~ 68 kDa, but its corresponding ligand had not been found yet (Fig. 5c), this indicated it should be a non-allosteric enzyme with auxiliary catalysis [29]. Geraniol isomerase was a pentameric protein with ~ 44 kDa (Fig. 5d), and its corresponding ligand was found as geraniol (ligand for nonmetallic ion not shown), indicating that the existence of geraniol was beneficial to the catalytic reaction [30]. In summary, these proteins were indispensable and directly participate in the regulation and biosynthesis of nerol.

The subsequent metabolism or transformation of nerol was catalyzed by geraniol 8-hydroxyase, alcohol dehydrogenase, and geraniol dehydrogenase. Among them, geraniol 8-hydroxyase was a monomeric protein of ~ 55 kDa [31], and its corresponding ligand had not been found yet (Fig. 5e). Alcohol dehydrogenase was also a monomeric protein with a molecular weight of ~ 39 kDa [32], its corresponding ligand was found as Zn^2+^ (Fig. 5f), indicating alcohol dehydrogenase could be bound and interacted with Zn^2+^, it would contribute to the formation of nerol. Geraniol dehydrogenase was a dimer protein with ~ 41 kDa [33], and its corresponding ligand had not been found (Fig. 5g), suggesting that it could be a non-allosteric enzyme with auxiliary catalysis. These evidences indicated that the generation of nerol was mainly attributed to the existence of corresponding enzymatic system and metabolic pathway in GXDK6. Furthermore, nerol was classified as a typical example of aromatic metabolites in GXDK6, why GXDK6 can maintain a long-term aroma production should be the contribution of various aromatic metabolites. Therefore, the biosynthesis mechanism of nerol will contribute to better understand of the aroma-producing mechanism of GXDK6.

## Conclusions

A novel aroma-producing *M. guilliermondii* GXDK6 was identified successfully by whole genome sequencing and metabolomics technology. GXDK6 showed high multi-stress-tolerant properties with acid–base, salty, and heavy-metal environments. Furthermore, GXDK6 could be fermented with 21 organic matters (including pentose and hexose) as the sole carbon sources and could produce abundant aromatic metabolites, such as alcohols, esters, acids, etc. The aroma-producing mechanism of nerol in GXDK6 had also been revealed. These findings indicated the *M. guilliermondii* GXDK6 has great potential value in the fermentation industry.

## Materials and methods

### Species and reagents

GXDK6 was isolated from the subtropical mangrove sediments in Beibu Gulf of South China Sea (21°29′25.74″N, 109°45′49.43″E) and deposited in the China General Microbiological Culture Collection Center (CGMCC) under CGMCC No. 16007.

The chemical reagents were analytical grade. Glucose, fructose, maltose, sorbitol, lactose, sucrose, mannitol, soluble starch, bran, cassava flour, and bagasse were purchased from Sigma-Aldrich, Inc. (Darmstadt, Germany). Yeast powder, agar powder, peptone, HCl, NaOH, NaCl, and KCl were purchased from Novagen (Darmstadt, Germany). Magnesium chloride, copper chloride, manganese chloride, and cobalt chloride were purchased from Sangon Biotech (Shanghai) Co., Ltd., (Shanghai, China).

### Physicochemical characterization of GXDK6

#### Colony characteristics and cell morphology

GXDK6 was incubated on the GBM agar plate at 30 ℃ for 48 h. The single colony was observed under the Olympus BX53M optical microscope (Olympus, Japan). The single colony without any chemical treatment was also scanned using a scanning electron microscope (TM3000; Olympus, Japan), with a scanning voltage set to 10 kV and magnification of 10, 000 folds.

#### Multi-stress-tolerant properties of GXDK6

The acid–base stress tolerance of GXDK6 was investigated in the pH range of 2.5–10.0. Salt tolerance was investigated by using NaCl, KCl, and MgCl_2_ in the concentration range of 12%–18%. Heavy-metal tolerance was investigated using seven heavy metals (i.e., Cd^2+^, Cu^2+^, Mn^2+^, Co^2+^, Ni^2+^, Cr^2+^, and Zn^2+^) in the concentration range of 0–5494 ppm. Temperature sensitivity was also investigated at 25–50 ℃. Three parallel experiments were performed, and the inoculation rate of GXDK6 was 2% (*v*:*v*). The growth of GXDK6 was evaluated using the turbidity method as reported by Irache et al. [34], with slight modifications.

#### Whole-genome sequencing analysis

GXDK6 was incubated by using an enrichment technique and GBM liquid medium (0.2% yeast extract, 0.2% beef extract, 0.5% polypeptone, 0.6% sucrose, pH 7.0). Both enrichment processes were carried out at 200 rpm and 30 ℃ for 8 h. The bacterial cells were then centrifuged at 8, 000 rpm and 4 °C for 10 min. Then, the cells were washed repeatedly with 0.1 mol/L PBS buffer.

The whole genomic DNA of GXDK6 was extracted using the CTAB method as reported by Van et al. [35], with slight modifications. The purity of the extracted DNA was verified by PCR (Polymerase Chain Reaction) and agarose gel electrophoresis [36]. The ITS gene was amplified by PCR using ITS1 (5′-TCCGTAGGTGAACCTGCGG-3′) /ITS4 universal primers (5′- TCCTCCGCTTATTGATATGC-3′) [37]. The ITS sequencing data of GXDK6 were deposited to the National Microbiology Data Center database (http://nmdc.cn) under the Accession Number of NMDCN000022O.

The whole-genome sequencing and analysis of GXDK6 were performed by the BGI Genomics Co., Ltd. (Shenzhen, China). An online software FastQC (http://www.bioinformatics.babraham.ac.uk/projects/fastqc) was used for the quality control of the second-generation sequencing downtime data. To determine the percentage of single-copy genes in the total single-copy genes, an online software BUSCO (http://busco.ezlab.org, v3.0.2) was conducted to complete the sequence comparison of the genome sequences. Hmmscan software [38] was adopted to predict the presence of Carbohydrate-Active enzymes (http://www.cazy.org) [39] genes in the genome sequence. The whole genome sequencing data of GXDK6 were deposited to the National Microbiology Data Center database (http://nmdc.cn) under the Accession Number of NMDC60014229.

### Aroma-producing properties of GXDK6

#### Fermentation of organic matter substrates

A total of 21 kinds of single organic matter (i.e., glucose, sucrose, fructose, xylose, xylan, sorbitol, raffinose, mannose, trehalose, cellulose, maltose, arabic candy, inulin, mannitol, sorbose, D-galactose, cellobiose, wheat bran, ethanol, succinic, and L-rhamnose; 2%) were added to the carbon-free medium [0.05% NaCl, 0.2% (NH_4_)_2_SO_4_, 0.05% K_2_HPO_4_, 0.05% KH_2_PO_4_, 0.02% MgSO_4‧_7H_2_O]. Among these compounds, ethanol was filtered through a 0.22-μm microporous membrane and then added to the sterilized (115 ℃ for 15 min) carbon-free medium. Then, 2% (*v:v*) of GXDK6 was inoculated in the above medium containing a single organic matter. To explore the ability of GXDK6 to ferment the single organic matters, experiments were performed on a rotary shaker at 200 rpm and 30 ℃ for 48 h.

#### Metabolomic analysis of GXDK6

GXDK6 was incubated in the medium with glucose, sucrose, fructose, or xylose as the sole carbon source for 72 h. Then, the fermentations were separated by centrifugation at 12, 000 × *g* for 10 min. The supernatants were then freeze-dried to powder by a refrigerated centrifugal concentrator (Shanghai, China). The powder samples were then derivatized for 120 min by using a 0.1 mg/mL methoxyamine hydrochloride-pyridine solution (Reagents for GC level) and alkylated for 120 min by using trifluoroacetamide (Reagent for GC level). When completed, the samples were centrifuged at 12, 000 × *g* for 10 min. The aromatic metabolites were detected and analyzed using GC–MS according to Pautova et al. [40], with slight modifications.

#### Annotation analysis of genes related to aroma production in GXDK6

According to the above metabolomics results of GXDK6, the genes related to aromatics metabolites were retrieved in the KEGG database (https://www.kegg.jp/kegg/genes.html). Sequence of the retrieved genes was then compared and annotated with the genomic data of GXDK6, which is used to screen the key genes of GXDK6 in regulating the expression of aromatic metabolites.

#### Data analysis of GXDK6

A phylogenetic tree was constructed using the MEGA7.0 software [41]. The whole genome sequencing results were analyzed using the FastQC software [21]. Data fitting and mapping analysis were performed using the Origin 9.0 software. Statistical analysis of other experimental data was performed using SPSS 17.0, and *P* values < 0.05 indicated significant differences.

## Supplementary Information

**Additional file 1.** Statistics of the sequencing data of GXDK6.

**Additional file 2.** Mass spectrum analysis of nerol when fermented with GXDK6 using glucose as the substrate.

**Additional file 3.** Gene annotation results related to the biosynthesis of nerol in GXDK6 (Identity ≥ 30%).

## Data Availability

All the data generated or analyzed during this study are included in the manuscript and its additional file.

## Abbreviations

GC–MS: Gas chromatography–mass spectrometry
*M. guilliermondii*: *Meyerozyma guilliermondii*
PCR: Polymerase chain reaction
ITS: Internal transcribed spacer
IPP: Isopentenyl diphosphate
DMAPP: Dimethylallyl diphosphate
NPP: Neryl diphosphate

## References

[1] Feng S, Huang M, Crane JH, Wang Y (2018). Characterization of key aroma-active compounds in lychee (*Litchi chinensis Sonn.*). J Food Drug Anal..

[2] Saeed Y, Faezeh D, Ali MB, Saeid H, Tumach Y, Khosro M (2018). Morphological, essential oil and biochemical variation of *Dracocephalum moldavica* L. populations. J Appl Res Med Aroma.

[3] Wang Y, Zeng X, Zhou Z, Xing K, Tessema A, Zeng H, Tian J (2015). Inhibitory effect of nerol against *Aspergillus niger* on grapes through a membrane lesion mechanism. Food Control.

[4] Tian J, Gan Y, Pan C, Zhang M, Wang X, Tang X, Peng X (2018). Nerol-induced apoptosis associated with the generation of ROS and Ca (2+) overload in saprotrophic fungus *Aspergillus flavus*. Appl Microbiol Biotechnol.

[5] Nedovic V, Gibson B, Mantzouridou TF, Bugarski B, Djordjevic V, Kalusevic A, Paraskevopoulou A, Sandell M, Smogrovicova D, Yilmaztekin M (2015). Aroma formation by immobilized yeast cells in fermentation processes. Yeast.

[6] Du Toit E, Vesterlund S, Gueimonde M, Salminen S (2013). Assessment of the effect of stress-tolerance acquisition on some basic characteristics of specific probiotics. Int J Food Microbiol.

[7] Bletz MC, Loudon AH, Becker MH, Bell SC, Woodhams DC, Minbiole KPC, Harris RN (2013). Mitigating amphibian chytridiomycosis with bioaugmentation: characteristics of effective probiotics and strategies for their selection and use. Ecol Lett.

[8] Srisukchayakul P, Charalampopoulos D, Karatzas KA (2018). Study on the effect of citric acid adaptation toward the subsequent survival of *Lactobacillus plantarum* NCIMB 8826 in low pH fruit juices during refrigerated storage. Food Res Int.

[9] Martínez Cruz P, Ibáñez AL, Monroy Hermosillo OA, Ramírez Saad HC (2012). Use of probiotics in aquaculture. ISRN Microbiology.

[10] Zong Z, Hua Q, Tong X, Li D, Wang C, Guo D, Liu Z (2019). Biosynthesis of nerol from glucose in the metabolic engineered *Escherichia coli*. Biores Technol.

[11] Coda R, Rizzello CG, Cagno RD, Trani A, Cardinali G, Gobbetti M (2013). Antifungal activity of *Meyerozyma guilliermondii*: Identification of active compounds synthesized during dough fermentation and their effect on long-term storage of wheat bread. Food Microbiol.

[12] Wah TT, Walaisri S, Assavanig A, Niamsiri N, Lertsiri S (2013). Co-culturing of *Pichia guilliermondii* enhanced volatile flavor compound formation by *Zygosaccharomyces rouxii* in the model system of Thai soy sauce fermentation. Int J Food Microbiol.

[13] Tayel AA, El-Tras WF, Moussa SH, El-Agamy MA (2013). Antifungal action of *Pichia anomala* against aflatoxigenic *Aspergillus flavus* and its application as a feed supplement. J Sci Food Agric.

[14] Kim WCKN (2009). Isolation, identification, and characterization of *Pichia guilliermondii* K123–1 and *Candida fermentati* SI, producing isoflavone β-glycosidase to hydrolyze isoflavone glycoside efficiently, from the Korean Traditional Soybean Paste. J Appl Biol Chem.

[15] Boretsky YR, Pynyaha YV, Boretsky VY, Fedorovych DV, Fayura LR, Protchenko O, Philpott CC, Sibirny AA (2011). Identification of the genes affecting the regulation of riboflavin synthesis in the flavinogenic yeast *Pichia guilliermondii* using insertion mutagenesis. FEMS Yeast Res.

[16] Kim HR, Kim JH, Bai DH, Ahn BH (2013). Microbiological characteristics of wild yeast strain *Pichia anomala* Y197–13 for Brewing Makgeolli. Mycobiology.

[17] Fernández PM, Martorell MM, Blaser MG, Ruberto LAM, de Figueroa LIC, Mac Cormack WP (2017). Phenol degradation and heavy metal tolerance of Antarctic yeasts. Extremophiles.

[18] Bhakta JN, Ohnishi K, Munekage Y, Iwasaki K, Wei MQ (2012). Characterization of lactic acid bacteria-based probiotics as potential heavy metal sorbents. J Appl Microbiol.

[19] Ndubuisi IA, Qin Q, Liao G, Wang B, Moneke AN, Ogbonna JC, Jin C, Fang W (2020). Effects of various inhibitory substances and immobilization on ethanol production efficiency of a thermotolerant *Pichia kudriavzevii*. Biotechnol Biofuels.

[20] Margulies M, Egholm M, Altman WE, Attiya S, Bader JS, Bemben LA, Berka J, Braverman MS, Chen Y, Chen Z (2005). Genome sequencing in open microfabricated high density picoliter reactors. Nature.

[21] Kim T, Seo HD, Hennighausen L, Lee D, Kang K (2018). Octopus-toolkit: a workflow to automate mining of public epigenomic and transcriptomic next-generation sequencing data. Nucleic Acids Res.

[22] Hunt M, Kikuchi T, Sanders M, Newbold C, Berriman M, Otto TD (2013). REAPR: a universal tool for genome assembly evaluation. Genome Biol.

[23] Lai YT, Cheng KC, Lai CN, Lai YJ (2019). Isolation and identification of aroma producing strain with esterification capacity from yellow water. PLoS ONE.

[24] Irma Isnafia A, Dyah Nurul A, Zakiah W, Cahyo B (2016). Physicochemical properties, fatty acid prfiles, and sensory characteristics of fermented beef sausage by probiotics *Lactobacillus plantarum* IIA-2C12 or *Lactobacillus acidophilus* IIA-2B4. J Food Sci.

[25] Liu Y, Cheng H, Liu H, Ma R, Ma J, Fang H (2019). Fermentation by multiple bacterial strains improves the production of bioactive compounds and antioxidant activity of goji juice. Molecules.

[26] Singh A, Vishwakarma V, Singhal B (2018). Metabiotics: the functional metabolic signatures of probiotics: current state-of-art and future research priorities-metabiotics: probiotics effector molecules. Adv Biosci Biotechnol.

[27] Wang M, Zhang X, Wang Y, Li Y, Chen Y, Zheng H, Ma F, Ma CW, Lu B, Xie Z (2018). Metabonomic strategy for the detection of metabolic effects of probiotics combined with prebiotic supplementation in weaned rats. RSC Adv.

[28] Nah J, Song SJ, Back K (2001). Partial characterization of farnesyl and geranylgeranyl diphosphatases induced in rice seedlings by UV-C irradiation. Plant Cell Physiol.

[29] Rai A, Smita SS, Singh AK, Shanker K, Nagegowda DA (2013). Heteromeric and homomeric geranyl diphosphate synthases from catharanthus roseus and their role in monoterpene indole alkaloid biosynthesis. Mol Plant..

[30] Marmulla R, Safaric B, Markert S, Schweder T, Harder J (2016). Linalool isomerase, a membrane-anchored enzyme in the anaerobic monoterpene degradation in *Thauera linaloolentis* 47Lol. BMC Biochem.

[31] Sintupachee S, Promden W, Ngamrojanavanich N, Sitthithaworn W, De-Eknamkul W (2015). Functional expression of a putative geraniol 8-hydroxylase by reconstitution of bacterially expressed plant CYP76F45 and NADPH-cytochrome P450 reductase CPR I from Croton stellatopilosus Ohba. Phytochemistry.

[32] Magnusson AO, Szekrenyi A, Joosten HJ, Finnigan J, Charnock S, Fessner WD (2019). nanoDSF as screening tool for enzyme libraries and biotechnology development. The FEBS Journal.

[33] Noge K, Kato M, Mori N, Kataoka M, Tanaka C, Yamasue Y, Nishida R, Kuwahara Y (2010). Geraniol dehydrogenase, the key enzyme in biosynthesis of the alarm pheromone, from the astigmatid mite iCarpoglyphuslactis/i (Acari: Carpoglyphidae). The FEBS Journal.

[34] Irache JM, Durrer C, Ponchel G, Duchêne D (1993). Determination of particle concentration in latexes by turbidimetry. Int J Pharm.

[35] van Burik JA, Schreckhise RW, White TC, Bowden RA, Myerson D (1998). Comparison of six extraction techniques for isolation of DNA from filamentous fungi. Med Mycol.

[36] Ehrhardt DW, Frommer WB (2012). New technologies for 21st century plant science. Plant Cell.

[37] Dupont D, Gaucherand P, Wallon M (2020). Fortuitous diagnosis of Trichomoniasis by PCR using panfungal primers. Int J Infect Dis.

[38] Lagesen K, Hallin P, Rodland EA, Staerfeldt HH, Rognes T, Ussery DW (2007). RNAmmer: consistent and rapid annotation of ribosomal RNA genes. Nucleic Acids Res.

[39] Lombard V, Golaconda RH, Drula E, Coutinho PM, Henrissat B (2014). The carbohydrate-active enzymes database (CAZy) in 2013. Nucleic Acids Res.

[40] Pautova AK, Bedova AY, Sarshor YN, Beloborodova NV (2018). Determination of aromatic microbial metabolites in blood serum by gas chromatography-mass spectrometry. J Anal Chem.

[41] Xia W, Zhao P, Yi Z, Cui Y (2017). Phylogenetic and specific sequence analysis of four paralogs in insect Aquaporins. Mol Med Rep.

