# Supplementary material for: Whole genome sequencing and metabolomics analyses reveal the biosynthesis of nerol in a multi-stress-tolerant Meyerozyma guilliermondii GXDK6
Source: Microb Cell Fact. 2021 Jan 3;20:4. doi: 10.1186/s12934-020-01490-2 (PMC7789178; doi:10.1186/s12934-020-01490-2)
Supplement: Supplementary file 1 — Additional file 1. Statistics of the sequencing data of GXDK6. [file 12934_2020_1490_MOESM1_ESM.docx]

**Supplemental Material**

**Title: Whole genome sequencing and metabolomics analyses reveal the biosynthesis of nerol in a multi-stress-tolerant *Meyerozyma guilliermondii* GXDK6**

**Authors:** Xueyan Mo ^1, a^, Xinghua Cai ^1, a^, Qinyan Hui ^1^, Huijie Sun ^1^, Ran Yu ^1^, Ru Bu ^1^, Bing Yan ^2^, Qian Ou ^1^, Quanwen Li ^1^, Sheng He ^3, *^, and Chengjian Jiang ^1, 2, *^

**Affiliation:**

(^1^State Key Laboratory for Conservation and Utilization of Subtropical Agro-bioresources, Guangxi Research Center for Microbial and Enzyme Engineering Technology, College of Life Science and Technology, Guangxi University, Nanning 530004, China.

^2^ Guangxi Key Lab of Mangrove Conservation and Utilization, Guangxi Mangrove Research Center, Guangxi Academy of Sciences, Beihai 536000, China.

^3^ Guangxi Birth Defects Prevention and Control Institute, Maternal and Child Health Hospital of Guangxi Zhuang Autonomous Region. Nanning 530033, China.)

**a:** These authors contributed equally to this work.

***: Corresponding author**

Tel: +86-771-3270736, Fax: +86-771-3237873

E-mail: jiangcj0520@vip.163.com (Chengjian Jiang); heshengbiol@163.com (Sheng He)

**Additional file 1**. Statistics of the sequencing data of GXDK6.

| Sample | Library | Reads number | Total bases (bp) | GC% | N% | Q_20_% | Q_30_% |
| --- | --- | --- | --- | --- | --- | --- | --- |
| Genome | PE400 | 8319572 | 2477073673 | 38.88 | 0.001 | 94.60 | 86.73 |

Note: N% indicates the percentage of fuzzy bases; Q_20_% indicates the percentage of bases with a base recognition accuracy rate is more than 99%; Q_30_% indicates the percentage of bases with a base recognition accuracy rate is more than 99.9%.
