# Supplementary material for: Whole genome sequencing and metabolomics analyses reveal the biosynthesis of nerol in a multi-stress-tolerant Meyerozyma guilliermondii GXDK6
Source: Microb Cell Fact. 2021 Jan 3;20:4. doi: 10.1186/s12934-020-01490-2 (PMC7789178; doi:10.1186/s12934-020-01490-2)

**Supplemental Material**

**Title: Whole genome sequencing and metabolomics analyses reveal the biosynthesis of nerol in a multi-stress-tolerant *Meyerozyma guilliermondii* GXDK6**

**Authors:** Xueyan Mo ^1, a^, Xinghua Cai ^1, a^, Qinyan Hui ^1^, Huijie Sun ^1^, Ran Yu ^1^, Ru Bu ^1^, Bing Yan ^2^, Qian Ou ^1^, Quanwen Li ^1^, Sheng He ^3, *^, and Chengjian Jiang ^1, 2, *^

**Affiliation:**

(^1^State Key Laboratory for Conservation and Utilization of Subtropical Agro-bioresources, Guangxi Research Center for Microbial and Enzyme Engineering Technology, College of Life Science and Technology, Guangxi University, Nanning 530004, China.

^2^ Guangxi Key Lab of Mangrove Conservation and Utilization, Guangxi Mangrove Research Center, Guangxi Academy of Sciences, Beihai 536000, China.

^3^ Guangxi Birth Defects Prevention and Control Institute, Maternal and Child Health Hospital of Guangxi Zhuang Autonomous Region. Nanning 530033, China.)

**a:** These authors contributed equally to this work.

***: Corresponding author**

Tel: +86-771-3270736, Fax: +86-771-3237873

E-mail: jiangcj0520@vip.163.com (Chengjian Jiang); heshengbiol@163.com (Sheng He)

**Additional file 2.** Mass spectrum analysis of nerol when fermented with GXDK6 using glucose as the substrate. Electron bombardment ionization source was used in mass spectrometry; the electron energy was 70 eV; the ion source temperature was 230 ℃; the scanning range was 35 ~ 350 m/z. The production of nerol was indicated by the black arrow.


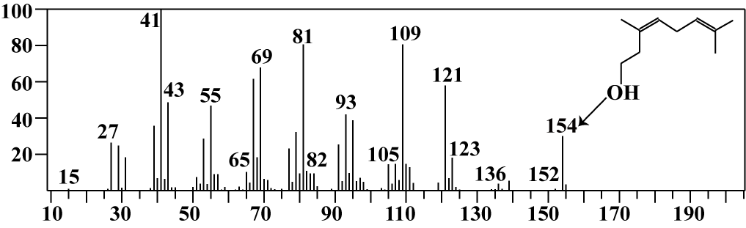

Supplement: Supplementary file 2 — Additional file 2. Mass spectrum analysis of nerol when fermented with GXDK6 using glucose as the substrate. [file 12934_2020_1490_MOESM2_ESM.docx]
