# Supplementary material for: Whole genome sequencing and metabolomics analyses reveal the biosynthesis of nerol in a multi-stress-tolerant Meyerozyma guilliermondii GXDK6
Source: Microb Cell Fact. 2021 Jan 3;20:4. doi: 10.1186/s12934-020-01490-2 (PMC7789178; doi:10.1186/s12934-020-01490-2)
Supplement: Supplementary file 3 — Additional file 3. Gene annotation results related to the biosynthesis of nerol in GXDK6 (Identity ≥ 30%). [file 12934_2020_1490_MOESM3_ESM.docx]

**Supplemental Material**

**Title: Whole genome sequencing and metabolomics analyses reveal the biosynthesis of nerol in a multi-stress-tolerant *Meyerozyma guilliermondii* GXDK6**

**Authors:** Xueyan Mo ^1, a^, Xinghua Cai ^1, a^, Qinyan Hui ^1^, Huijie Sun ^1^, Ran Yu ^1^, Ru Bu ^1^, Bing Yan ^2^, Qian Ou ^1^, Quanwen Li ^1^, Sheng He ^3, *^, and Chengjian Jiang ^1, 2, *^

**Affiliation:**

(^1^State Key Laboratory for Conservation and Utilization of Subtropical Agro-bioresources, Guangxi Research Center for Microbial and Enzyme Engineering Technology, College of Life Science and Technology, Guangxi University, Nanning 530004, China.

^2^ Guangxi Key Lab of Mangrove Conservation and Utilization, Guangxi Mangrove Research Center, Guangxi Academy of Sciences, Beihai 536000, China.

^3^ Guangxi Birth Defects Prevention and Control Institute, Maternal and Child Health Hospital of Guangxi Zhuang Autonomous Region. Nanning 530033, China.)

**a:** These authors contributed equally to this work.

***: Corresponding author**

Tel: +86-771-3270736, Fax: +86-771-3237873

E-mail: jiangcj0520@vip.163.com (Chengjian Jiang); heshengbiol@163.com (Sheng He)

**Additional file 3.** Gene annotation results related to the biosynthesis of nerol in GXDK6 (Identity ≥ 30%).

| Species | Description | Gene | subject ID | Identity/% | E-vaule |
| --- | --- | --- | --- | --- | --- |
| P00817 | Inorganic pyrophosphatase | IPP1 | scaffold2.t446 | 75.2 | 3.80E-132 |
| P39940 | E3 ubiquitin-protein ligase RSP5 | RSP5 | scaffold2.t650 | 74.4 | 0.00E+00 |
| G8ZRX5 | (2E,6E)-farnesyl diphosphate synthase | TDEL0C03780 | scaffold2.t733 | 61.5 | 8.00E-124 |
| Q6L9N0 | (2E,6E)-farnesyl diphosphate synthase (Fragment) | ERG20 | scaffold2.t733 | 61.4 | 2.70E-77 |
| A0A0U1M8Z0 | Alcohol dehydrogenase | adhA | scaffold2.t903 | 61.4 | 1.50E-117 |
| A0A384JYI5 | Isopentenyl-diphosphate Delta-isomerase | Bcidi1 | scaffold3.t708 | 61.3 | 2.80E-78 |
| Q10132 | Isopentenyl-diphosphate Delta-isomerase | idi1 | scaffold3. t708 | 61 | 1.10E-79 |
| W0T5C0 | (2E,6E)-farnesyl diphosphate synthase | FPS1 | scaffold2.t733 | 60.9 | 5.90E-119 |
| P49349 | Farnesyl pyrophosphate synthase | FPS1 | scaffold2.t733 | 60.7 | 1.70E-118 |
| G0VAP1 | (2E,6E)-farnesyl diphosphate synthase | NCAS0B08330 | scaffold2.t733 | 60.7 | 3.70E-121 |
| A2R9I3 | Alcohol-dehydrogenase adhA from patent WO8704464-A-Aspergillus niger | adhA | scaffold2.t903 | 59.9 | 1.20E-116 |
| A0A0W0DX29 | (2E,6E)-farnesyl diphosphate synthase | ERG20 | scaffold2.t733 | 59.5 | 5.40E-120 |
| Q6FLV7 | (2E,6E)-farnesyl diphosphate synthase | ERG20 | scaffold2.t733 | 59.5 | 5.40E-120 |
| H2AXH6 | (2E,6E)-farnesyl diphosphate synthase | KAFR0G00430 | scaffold2.t733 | 59.5 | 2.10E-116 |
| A0A367XMZ4 | (2E,6E)-farnesyl diphosphate synthase | ERG20 1 | scaffold2.t733 | 59.3 | 5.40E-120 |
| A0A1D8PH78 | (2E,6E)-farnesyl diphosphate synthase | ERG20 | scaffold2.t733 | 59 | 2.70E-119 |
| A0A367YPB6 | (2E,6E)-farnesyl diphosphate synthase | ERG20 0 | scaffold2.t733 | 59 | 2.00E-119 |
| A0A6A5PW03 | (2E,6E)-farnesyl diphosphate synthase (Fragment) | ERG20 | scaffold2.t733 | 58.7 | 9.50E-117 |
| P08524 | Farnesyl pyrophosphate synthase | ERG20 | scaffold2.t733 | 58.5 | 4.70E-116 |
| C7GRZ5 | (2E,6E)-farnesyl diphosphate synthase | ERG20 | scaffold2.t733 | 58.5 | 4.70E-116 |
| G2WGK6 | (2E,6E)-farnesyl diphosphate synthase | K7 ERG20 | scaffold2.t733 | 58.5 | 4.70E-116 |
| A6ZQG5 | (2E,6E)-farnesyl diphosphate synthase | ERG20 | scaffold2.t733 | 58.5 | 6.20E-116 |
| A0A6C1DTF3 | (2E,6E)-farnesyl diphosphate synthase | ERG20 1 | scaffold2.t733 | 58.5 | 8.10E-116 |
| A0A167F685 | (2E,6E)-farnesyl diphosphate synthase | ERG20 | scaffold2.t733 | 58.3 | 3.60E-72 |
| G3AFE8 | Trehalose-6-phosphate phosphatase | TPS2 | scaffold6.t159 | 58.1 | 5.80E-280 |
| G8ZMP3 | Isopentenyl-diphosphate Delta-isomerase | TDEL0A05550 | scaffold3.t708 | 57.8 | 6.80E-86 |
| A3LXL1 | Threalose-6-phosphate phosphatase | TPS2 | scaffold6.t159 | 57.7 | 1.80E-281 |
| A0A367XUK0 | Trehalose-phosphatase | TPS2 0 | scaffold6.t159 | 57.5 | 3.70E-282 |
| A0A367J0G2 | (2E,6E)-farnesyl diphosphate synthase (Fragment) | ERG20 1 | scaffold2.t733 | 57.4 | 1.20E-116 |
| A0A367IJN0 | (2E,6E)-farnesyl diphosphate synthase (Fragment) | ERG20 1 | scaffold2.t733 | 57.4 | 6.40E-101 |
| A0A367KM35 | (2E,6E)-farnesyl diphosphate synthase | ERG20 2 | scaffold2.t733 | 57.4 | 2.30E-115 |
| G1UAE0 | Trehalose-6-phosphate phosphatase | TPS2 | scaffold6.t159 | 57.3 | 1.00E-279 |
| Q5AI14 | Trehalose-phosphatase | TPS2 | scaffold6.t159 | 57.3 | 1.00E-279 |
| A3LU92 | (2E,6E)-farnesyl diphosphate synthase | ERG20 | scaffold2.t733 | 57.1 | 6.80E-115 |
| G0VCF4 | Isopentenyl-diphosphate Delta-isomerase | NCAS0C01740 | scaffold3.t708 | 57 | 3.40E-85 |
| A0A6C1EAJ1 | (2E,6E)-farnesyl diphosphate synthase | ERG20 2 | scaffold2.t733 | 57 | 3.40E-114 |
| A0A367J7H5 | (2E,6E)-farnesyl diphosphate synthase | ERG20 2 | scaffold2.t733 | 56.9 | 6.20E-116 |
| A0A367Y5C2 | Trehalose-phosphatase | TPS2 1 | scaffold6.t159 | 56.8 | 1.60E-277 |
| F2QPA2 | (2E,6E)-farnesyl diphosphate synthase | ERG20 | scaffold2.t733 | 56.6 | 9.60E-109 |
| K5XKI8 | Isopentenyl-diphosphate Delta-isomerase | AGABI1DRAFT 110499 | scaffold3.t708 | 56.4 | 2.10E-65 |
| A0A1B2J7S2 | (2E,6E)-farnesyl diphosphate synthase | ERG20 | scaffold2.t733 | 56.1 | 2.40E-107 |
| A0A5Q4BYB7 | Adhesion and hyphal regulator 1 | AHR1 | scaffold1.t1055 | 55.6 | 3.20E-07 |
| A0A4R8QCN2 | Adhesion and hyphal regulator 1 | AHR1 | scaffold1.t1055 | 55.6 | 3.30E-07 |
| A0A6G1LW41 | Farnesyl pyrophosphate synthetase | ERG20 | scaffold2.t733 | 55.4 | 5.50E-109 |
| W6PZR5 | (2E,6E)-farnesyl diphosphate synthase | FPPS | scaffold2.t733 | 55.1 | 7.50E-82 |
| A2QRX8 | Isopentenyl-diphosphate Delta-isomerase | An08g07570 | scaffold3.t708 | 54.8 | 1.20E-79 |
| Q92218 | (2E,6E)-farnesyl diphosphate synthase (Fragment) | FPPS | scaffold2.t733 | 54.7 | 1.30E-63 |
| A0A4Z0XAL0 | (2E,6E)-farnesyl diphosphate synthase | ERG20 | scaffold2.t733 | 54.5 | 5.20E-107 |
| Q5A4F3 | Adhesion and hyphal regulator 1 | AHR1 | scaffold6.t54 | 54.5 | 1.90E-06 |
| A5AAE1 | (2E,6E)-farnesyl diphosphate synthase | An02g10350 | scaffold2.t733 | 54.1 | 1.00E-102 |
| A0A4C2DYJ2 | (2E,6E)-farnesyl diphosphate synthase | ERG20 | scaffold2.t733 | 54 | 1.60E-108 |
| F9XIB9 | (2E,6E)-farnesyl diphosphate synthase | ERG20 | scaffold2.t733 | 54 | 7.90E-108 |
| A0A507R2F8 | (2E,6E)-farnesyl diphosphate synthase | ERG20 | scaffold2.t733 | 53.3 | 2.30E-99 |
| A0A2Z6EYV1 | (2E,6E)-farnesyl diphosphate synthase | FDPS | scaffold2.t733 | 53.3 | 2.30E-102 |
| A0A1B2J9T3 | BA75 01812T0 | TPS2 | scaffold6.t159 | 53.2 | 2.20E-252 |
| F2QLT2 | Trehalose-6-P synthase/phosphatase complex subunit | TPS2 | scaffold6.t159 | 53.1 | 2.70E-250 |
| Q92250 | Farnesyl pyrophosphate synthase | fpp | scaffold2.t733 | 52.9 | 2.90E-102 |
| A0A2N9B027 | NADP-dependent alcohol dehydrogenase C 2 | adhC2 1 | scaffold7.t431 | 52.8 | 8.70E-10 |
| A0A161HGJ7 | (2E,6E)-farnesyl diphosphate synthase | ERG20 | scaffold2.t733 | 52.8 | 8.30E-30 |
| A0A2N6NMW7 | (2E,6E)-farnesyl diphosphate synthase | FPPS | scaffold2.t733 | 52.5 | 9.80E-98 |
| A0A376MRZ5 | Putative zinc-binding dehydrogenase | yahK 1 | scaffold7.t431 | 52.5 | 1.50E-12 |
| D3K2G1 | Trehalose-phosphate synthase (Fragment) | TPS2 | scaffold6.t159 | 52.4 | 2.30E-246 |
| A0A3G2S9D0 | (2E,6E)-farnesyl diphosphate synthase | FPS1 | scaffold2.t733 | 52.1 | 1.80E-102 |
| Q08645 | Folylpolyglutamate synthase | MET7 | scaffold2.t721 | 52.1 | 1.80E-138 |
| A0A4S3JVH3 | (2E,6E)-farnesyl diphosphate synthase | ERG20 2 | scaffold2.t733 | 52 | 3.60E-100 |
| A0A0F7TT74 | (2E,6E)-farnesyl diphosphate synthase | FPPS | scaffold2.t733 | 52 | 7.50E-98 |
| A0A177A008 | (2E,6E)-farnesyl diphosphate synthase | ERG20 | scaffold2.t733 | 51.7 | 1.40E-99 |
| A0A1B8GLD3 | (2E,6E)-farnesyl diphosphate synthase | ERG20 | scaffold2.t733 | 51.7 | 2.70E-100 |
| O14230 | Farnesyl pyrophosphate synthase | fps1 | scaffold2.t733 | 51.2 | 9.80E-98 |
| G3FFZ9 | Calreticulin (Fragment) | crt1 | scaffold1.t106 | 51.1 | 6.90E-36 |
| G3FFZ4 | Calreticulin (Fragment) | crt1 | scaffold1.t106 | 51.1 | 6.90E-36 |
| G3FFY7 | Calreticulin (Fragment) | crt1 | scaffold1.t106 | 51.1 | 6.90E-36 |
| G3FFZ0 | Calreticulin (Fragment) | crt1 | scaffold1.t106 | 51.1 | 4.10E-36 |
| A0A2Z6EYU3 | Diphosphomevalonate decarboxylase | MVAD | scaffold1.t969 | 51 | 6.40E-100 |
| A0A0H5CGR1 | TPS2 protein | TPS2 | scaffold6.t159 | 50.9 | 3.80E-244 |
| A0A559LXD2 | (2E,6E)-farnesyl diphosphate synthase | FPPS | scaffold2.t733 | 50.9 | 1.70E-97 |
| H2AZD3 | Isopentenyl-diphosphate Delta-isomerase | KAFR0H02800 | scaffold3.t708 | 50.9 | 1.10E-73 |
| A0A024F8C7 | Trehalose-6-phosphate synthase2 | tps2 | scaffold2.t595 | 50.5 | 1.20E-155 |
| W0T6Q3 | Trehalose-phosphatase | TPS2 | scaffold6.t159 | 50.4 | 6.30E-226 |
| Q92235 | Farnesyl pyrophosphate synthase | FPPS | scaffold2.t733 | 50.3 | 7.00E-96 |
| A0A0U5H5Z3 | Putative BZIP transcription factor JlbA/IDI-4 | ASPCAL12563 | scaffold2.t428 | 50 | 4.80E-04 |
| P48743 | RFX-like DNA-binding protein RFX1 | RFX1 | scaffold7.t386 | 50 | 7.80E-05 |
| A0A2I7G3B3 | Geraniol dehydrogenase (NAD+) | ADH2 | scaffold3. t238 | 41.5 | 1.90E-76 |
| A0A084G0S1 | 8-hydroxygeraniol dehydrogenase | SAPIO | scaffold7. t431 | 41.4 | 5.70E-70 |
| A0A2H5ZL72 | Geranial dehydrogenase | geoB | scaffold3. t157 | 40.6 | 2.30E-96 |
| Q2KNL6 | Alcohol dehydrogenase (NADP+) | GEDH1 | scaffold7. t431 | 36.5 | 7.20E-57 |
